# Effect of Bonding Material and Fixing Techniques on the Calibration of Copper–Indium Bimetallic-Coated FBG-Based Cryogenic Temperature Sensors for Aerospace Applications

**DOI:** 10.3390/s26185773

**Published:** 2026-09-11

**Authors:** Abrham Kassa Alem, Adriana Morana, Sylvain Girard, Jean-Pierre Chatelon, Peiqing Yu, Minh Chau Phan-Huy, Wendy Tomboza, Santerelli Falzon Tetsing Talla, Francis Vocanson, Fiammetta Fricano, Arnaud Meyer, Jean-Yves Michalon, Didier Pohl, Aziz Boukenter, Youcef Ouerdane, Emmanuel Marin

**Affiliations:** 1 Laboratoire Hubert Curien, UMR CNRS 5516, Université Jean Monnet, 42000 Saint-Etienne, France; adriana.morana@univ-st-etienne.fr (A.M.); sylvain.girard@univ-st-etienne.fr (S.G.); jean.pierre.chatelon@univ-st-etienne.fr (J.-P.C.); francis.vocanson@univ-st-etienne.fr (F.V.); fiammetta.fricano@univ-st-etienne.fr (F.F.); arnaud.meyer@univ-st-etienne.fr (A.M.); jean.yves.michalon@univ-st-etienne.fr (J.-Y.M.); aziz.boukenter@univ-st-etienne.fr (A.B.); ouerdane@univ-st-etienne.fr (Y.O.); 2 Institut Universitaire de France (IUF), Ministère de l’Enseignement Supérieur et de la Recherche, 1 rue Descartes, 75005 Paris, France; 3 Physics-Based Research on Wave-Matter Interaction, Sensing and Monitoring (PRISM), Safran Tech, Rue des Jeunes Bois, Châteaufort, 78114 Magny-Les-Hameaux, France; peiqing.yu@safrangroup.com (P.Y.); minh-chau.phan-huy@safrangroup.com (M.C.P.-H.); wendy.tomboza@safrangroup.com (W.T.); didier.pohl@safrangroup.com (D.P.)

**Keywords:** cryogenic temperature sensor, fiber Bragg grating, thin-film coating, electrodeposition

## Abstract

To enable fiber Bragg gratings (FBGs) to operate effectively as cryogenic temperature sensors, metals with high thermal expansion coefficients should be deposited onto the surface of an optical fiber. In this work, targeting aerospace applications, a copper–indium bimetallic-coated FBG designed for operation at around 850 nm was manufactured using sputtering and electrodeposition techniques. Furthermore, the influence of bonding materials, specifically Kapton tape and Apiezon N grease, as well as fixation methods, on the performance of the coated FBG was investigated during cryogenic temperature calibration. The results demonstrate that with Kapton tape bonding, the Bragg wavelength shift (BWS) was approximately 1.6 times greater than that obtained with Apiezon N grease at 77 K during sensor calibrations. However, in order to have efficient thermal coupling between the sample holder and the metal-coated sensor in harsh environments such as cryogenic and vacuum environments, the optimal choice of bonding material as well as its architecture is mandatory to achieve high sensor sensitivity. Therefore, this study gives insights into the fact that metal-coated FBGs’ calibration accuracy and real-time stability during sensor deployments can be affected by the fixing techniques and the bonding materials used.

## 1. Introduction

Temperature monitoring of cryogenic liquid storage tanks, which are used as coolants or fuels in many aerospace applications, is crucial to ensure that all the critical systems operate within the safe operating range and to take any preventive and corrective actions when abnormalities occur. Common abnormalities such as cryogen leaks and uncontrolled state changes can lead to serious system failures as well as significant economic losses. The most common cryogens in the aerospace domain are liquid hydrogen, oxygen, nitrogen and helium. Additionally, some of these cryogens are used in superconducting magnets for applications such as magnetic resonance imaging and high-energy physics facilities [1]. In these application domains, the temperature of superconducting magnets and cryogenic storage tanks must be continuously monitored using mostly either electronic or optical sensors. Typically, fiber Bragg gratings (FBGs) can be excellent candidates for designing point or quasi-distributed temperature sensors. An FBG corresponds to a periodic modulation of the fiber core’s refractive index along the propagation axis. The refractive index modulation is achieved using a sufficiently intense laser beam and interferometry or point-by-point inscription techniques [2,3,4,5,6,7,8,9]. Compared with electronics-based alternative-sensing technologies, FBGs offer inherent multiplexing capability, immunity to electromagnetic perturbations and lower costs [5,10].

Environmental perturbation parameters such as temperature or strain applied to an FBG alter the effective refractive index of the guided mode ($n_{eff}$) and the grating period (Λ). Both the effective refractive index and the grating period are temperature- and strain-dependent. The Bragg wavelength ($\lambda_{B})\;$ of the FBG corresponds to the specific wavelength at which the light will be selectively reflected by the periodic structure. It is determined by the constructive interference condition and is given by Equation (1) [4,5]:

(1) $$\lambda_{B}=2n_{eff}\Lambda$$

Environmental perturbations on the FBG will cause a blue or red shift in $\lambda_{B}.\;$ After calibration, the evolution of the Bragg wavelength shift (BWS) can be used to monitor the real-time temperature or strain applied to the FBG. For example, around room temperature (RT), the strain and temperature sensitivities of a bare FBG inscribed within a germanosilicate single-mode optical fiber are about 1.2 pm/με and 10 pm/K, respectively, at ~1.5 μm [4,5]. However, due to a drastic reduction in both the thermal expansion and thermo-optic coefficients of silica at cryogenic temperatures, these bare FBGs are not efficient sensors at these temperatures. To overcome this intrinsic limitation, it is possible, for example, to bond the FBG to a high-thermal-expansion-coefficient substrate using cryo-compatible glues. Another solution is to develop functional coatings on the surface of the bare FBG with high-thermal-expansion-coefficient metals or polymers using micromanufacturing techniques to enhance its thermal sensitivity at these extremely low temperatures [11,12,13,14,15,16].

Despite the higher sensitivities provided by polymer-coated FBGs [11,15], functional material coating of FBGs with either single- or bimetallic-based coatings can be a better approach for very low cryogenic temperatures in terms of stability and long-term reliability [12,14,16]. As an example, in previous studies, aluminum, copper, lead and indium depositions were investigated as FBG coating materials, using aluminum as a primary coating layer on the surface of silica. In particular, it has been shown that FBGs coated with indium and lead significantly extend the temperature detection range and increase the performance of this sensor down to liquid-helium temperatures [13,16].

The way to enhance an FBG’s thermal sensitivity through these coatings or packaging choices is twofold. First, the intrinsic temperature response of the FBG is governed by the thermo-optic effect and the thermal expansion of the fiber. However, at very low cryogenic temperatures, this intrinsic thermal response of the silica is almost negligible. Second, additional sensitivity is achieved through temperature-induced strain transferred from the coating material. As the temperature varies, the added coating undergoes differential thermal expansion, generating thermally induced strain. This strain is subsequently transferred to the FBG, resulting in additional modifications of both the effective refractive index of the guided mode and the grating period. Consequently, these combined effects lead to a larger BWS. For a coated FBG, the thermal sensitivity (K_T_), which is defined by the derivative of the Bragg wavelength with respect to temperature for such coated FBGs, can be modeled by Equation (2) [17].

K_T_ = [(1 − p_e_) (α_w_ − α_f_) + (α_f_ − ξ)] λ_B_ (2)

where p_e_, *ξ*, *α*_w_, *α*_f_, and *λ*_B_ are the elasto-optic coefficient of silica, thermo-optic coefficient of silica, weighted mean thermal expansion coefficient of silica and the coating, silica thermal expansion coefficient and Bragg wavelength respectively.

The performances of such coated FBGs can be highly affected by the coating process parameters, such as the adhesion of the coating material to silica, the coating thickness, and the choices made regarding material layer combinations and compatibilities [18,19]. Furthermore, even the choice of Bragg wavelengths, e.g., operating at around 1.5 µm or around 850 nm, can affect the sensor sensitivity. Moreover, a lack of standardized bonding materials and fixing techniques during sensor calibration and deployment can negatively affect the calibration accuracy and thermal stability [20]. In this work, copper–indium bimetallic-coated FBGs were fabricated using a two-stage micromanufacturing process. The influence of the bonding materials on the sensing performance of this class of coated FBGs at an 850 nm interrogation wavelength was systematically investigated, particularly in the context of their fixing to a cryostat’s sample holder during their calibration. The aim was to demonstrate an industrially viable, miniaturized sensing chain with a high temperature resolution (at least 0.1 K). Ultimately, this approach offers a lighter, more cost-effective, and compact alternative to traditional 1550 nm sensing chains. In general, these miniaturized bimetallic-coated sensors facilitate versatile installation via soldering, adhesives, or freestanding configuration, thereby enhancing sensitivities as well as avoiding the larger footprint and bulk typically associated with substrate-bonded alternatives.

## 2. Materials and Methods

### 2.1. FBG Functional Material Coating

**Figure 1:**
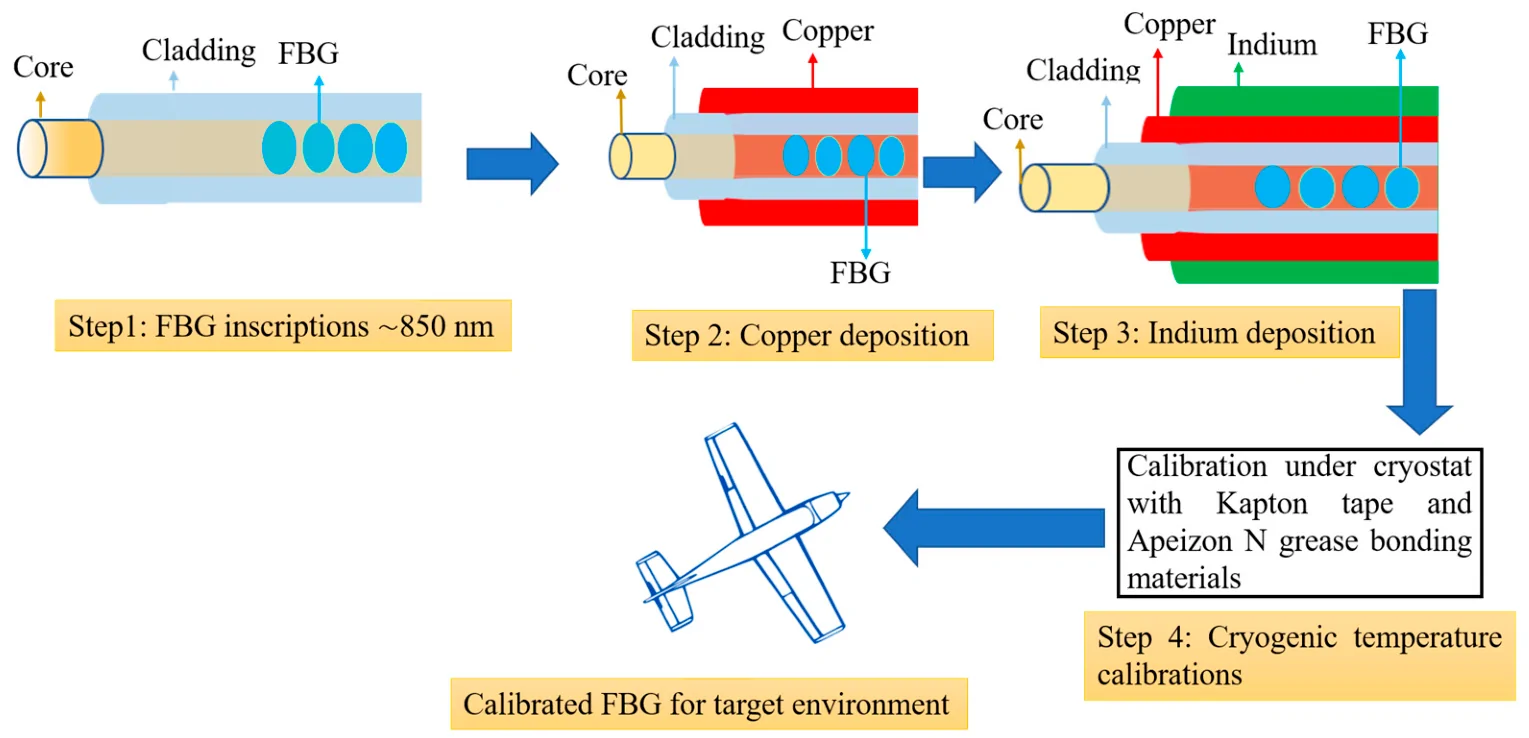
Schematic description of the different manufacturing steps for copper–indium bimetallic-coated FBG sensor development. Colors in the schematic coded in blue, red, and green represent FBG, copper and indium coatings respectively.

The 5 mm long FBG sensors were inscribed into a hydrogen-loaded germanium-doped SM800 fiber from Thorlabs (Newton, NJ, USA) using a UV cw laser at 244 nm and the Lloyd mirror interferometry technique [4,5]. The laser power used for the inscriptions was ~121 mW, and the FBGs’ operating Bragg wavelength was chosen to be around 850 nm with a ~0.3 μm grating period. Moreover, to enhance the germanosilicate optical fiber’s photosensitivity, the sample was loaded with hydrogen for one week at 110 bars and room temperature [5,21]. The FBGs used for this work were coated with 5 µm and 585 µm thicknesses of copper and indium bimetallic layers using sputtering- and electrodeposition-based micromanufacturing techniques respectively. The details of the micro-nanofabrication processes utilized in this work, as well as the choice of operating wavelength and interrogator used for aerospace applications [22,23], were reported in a previous work [24]. Furthermore, the electrolyte chemical composition for the secondary indium coating was adapted from [25], and a custom setup with a single platinum anode electrode was developed. The different manufacturing steps used to produce the copper–indium bimetallic-coated FBG sensors are schematically illustrated in Figure 1.

### 2.2. Effects of Bonding Materials and FBG Fixing Techniques on Coated FBG Calibrations

**Figure 2:**
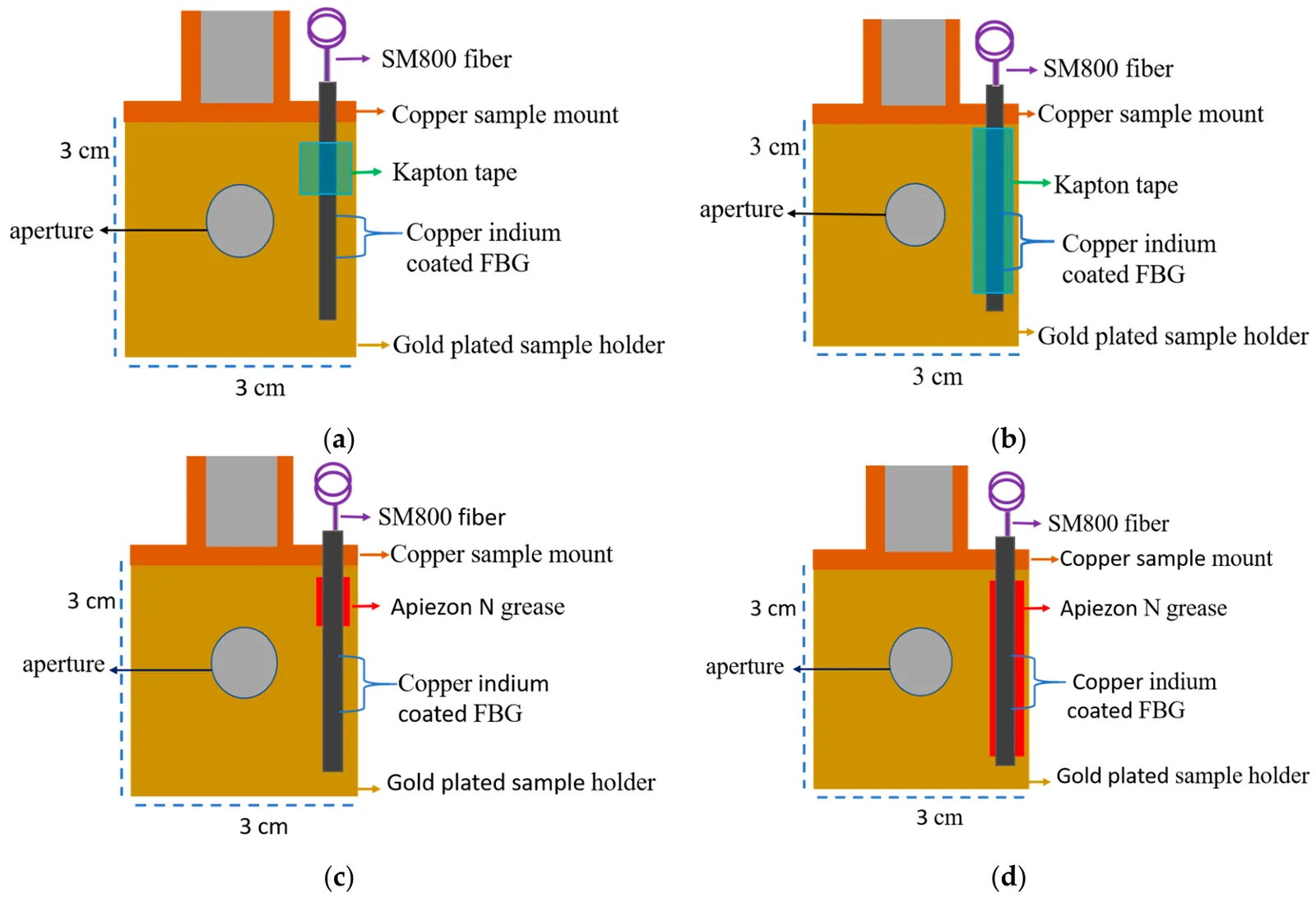
(**a**) Kapton tape one-sided bonding, (**b**) Kapton tape full bonding, (**c**) Apiezon N thermal grease one-sided bonding and (**d**) Apiezon N thermal grease full bonding. The sample holder also has an aperture.

Directly bonding FBG sensors to high-temperature superconducting coils with adhesives is a common, non-destructive method to ensure high sensitivity during cryogenic temperature monitoring [14]. Additionally, it is a cost-effective technique that does not require any further machining to bond the sensors to other target cryogenic structures. Bonding materials such as Apiezon N thermal grease and Stycast (for permanent bonding) can be used for such applications [14]. In this work, Kapton tape and Apiezon N grease were chosen as they can be used to temporarily attach an FBG to a cryostat’s sample holder to ensure optimal thermal contact during calibration within the vacuum and cryogenic environments, aiming to approximately simulate real-case scenarios. For example, Kapton tape was demonstrated to improve the real-time stability of FBG-based sensors for space applications [20]. During testing and calibration, in order to optimize thermal coupling, these materials can be applied along the metallized section of the optical fiber, including the FBGs (hereafter referred to as full bonding) or only along a partial section of it, without any bonding on the FBG (hereafter referred to as one-sided bonding). The schematic in Figure 2 details the various physical arrangements and connections investigated involving the ST 100 optical cryostat (Janis research company, Woburn, MA, USA, [now part of Lake Shore Cryotronics], Westerville, OH, USA) sample mount, sample holder, and the metalized FBGs, as well as their fixing methods with different bonding materials. Finally, the fiber is connected through a vacuum feedthrough to collect the reflection measurement signal. Moreover, careful prior calibration is a crucial step for evaluating the sensor performance before the final deployment on the target structures. For the sake of comparison, during the investigation on the effect of bonding materials and fixing techniques, all the testing conditions, such as the Apiezon N thermal grease thickness (~0.9 mm), thermal stabilization time and thickness of both the first copper layer and the second indium one, were kept identical. However, to investigate the effect of the microfabrication process, specifically electrodeposition, on the FBG’s spectral quality, bimetallic-coated FBGs with different thicknesses of indium as a secondary layer were also manufactured and characterized.

**Figure 3:**
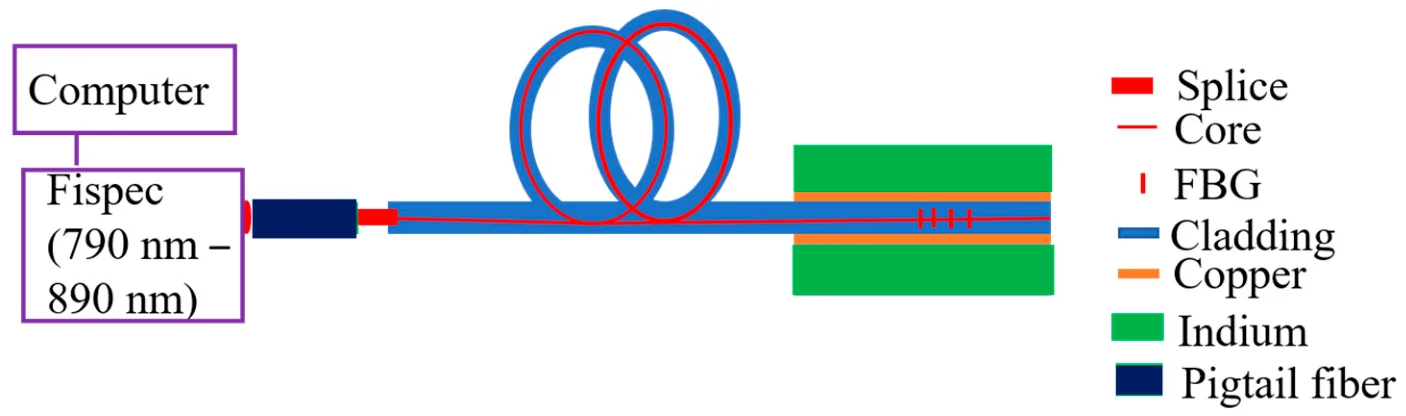
Schematics for copper–indium bimetallic-coated FBGs in reflection configuration setup.

Thermal-cycling tests and sensor calibrations were carried out using the cryostat, cooled by liquid nitrogen. A LakeShore 335 temperature controller and a silicon (Si) diode sensor (Lake Shore Cryotronics, Westerville, OH, USA) provided the reference temperature, in steps of 10 K (20 K for a few measurements). The sensor was allowed to thermally stabilize for 30 min before a 10 min measurement window began at each temperature step. During this window, the copper–indium bimetallic-coated FBG spectra and the corresponding Bragg wavelength were measured using a Fisens Fispec interrogator [22,23] in a reflection setup configuration, as shown in the schematics in Figure 3. A vacuum level between 2 × 10^−6^ mbar and 4 × 10^−7^ mbar was achieved using cascaded electromechanical pumps. Finally, the spectra were recorded every second with an integration time of 100 ms, and the final Bragg wavelength for each temperature was determined by using a Gaussian-based peak-tracking algorithm and averaging the Bragg wavelength values obtained over the measurement period. The standard deviation was taken as the error bar of the measurement. For the detailed data analysis, the Bragg wavelength shift (BWS), which is now defined as the change in the Bragg wavelength relative to its value at room temperature (RT, specifically 300 K), was calculated.

## 3. Results

### 3.1. Effect of Metallization Process on Quality of FBG Spectra

The evolution of each produced FBG spectrum was monitored before and after each of the two different successive steps of the metal deposition processes: first, the copper coating and then second, the indium coating, as shown in Figure 1. The objective was to highlight the influences of both processes on the FBG’s properties, such as the quality of its spectral shape and the evolution of its Bragg wavelength. These results are discussed in the following Section 3.1.1 and Section 3.1.2.

#### 3.1.1. Effect of Sputtering Process on FBG Spectra

**Figure 4:**
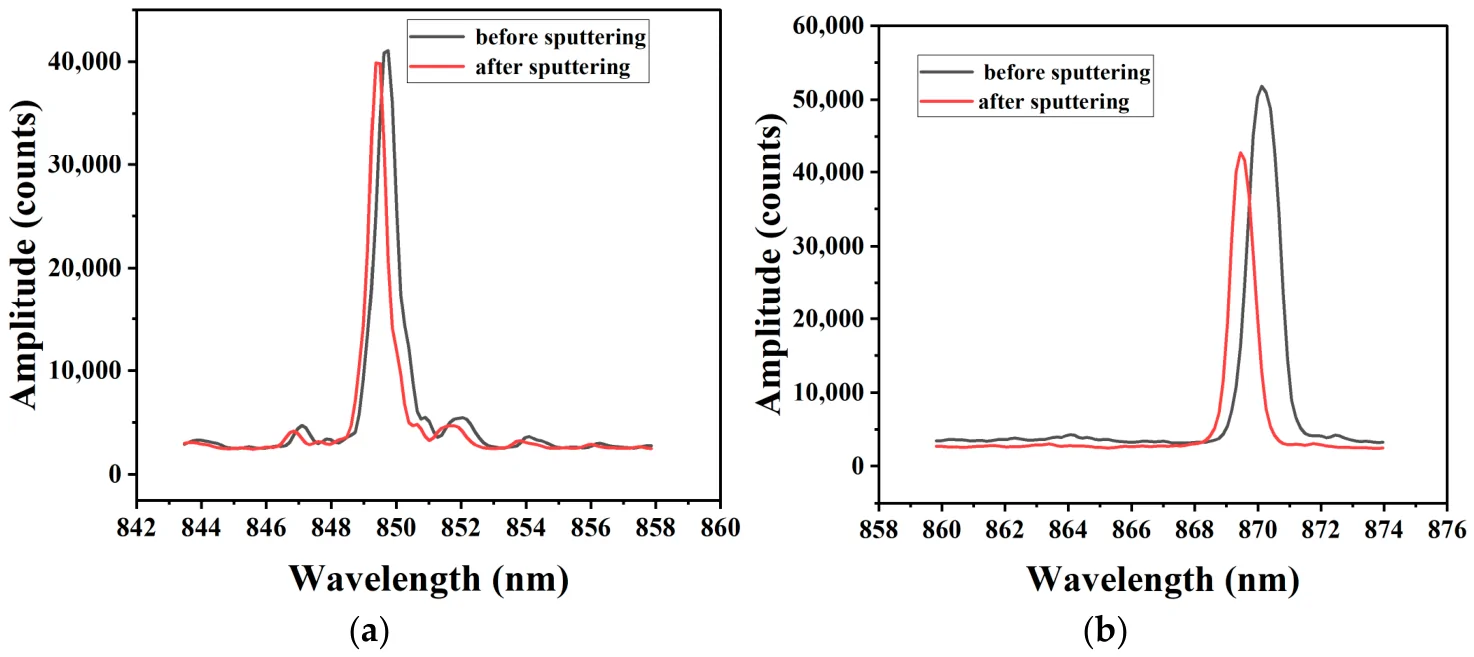
Effect of sputtering process on the quality of the FBG spectra for (**a**) a pre-annealed FBG at 290 °C for 3 h prior to the sputtering step and (**b**) an FBG without any pre-annealing prior to the sputtering step.

The sputtering process was used to deposit a 5 µm thick Cu primary coating, resulting in an average blue shift of 0.57 nm in the Bragg wavelengths of eight different FBGs, with a standard deviation of 0.15 nm. Typical spectra are given in Figure 4a,b for two of these FBGs. The blue BWS exhibited by all the FBGs can be explained by the erasure of the unstable part of our UV type I FBGs [21] because of a thermal treatment related to the process temperature reaching a maximum of around 250 °C. Another explanation is that this could have resulted from an applied overall longitudinal compressive force during the copper metal film growth [26]. The blueshift for the first spectrum was due to the compressive strain during the metallization process, as the unstable gratings were already completely erased by thermal annealing, validated by a study on the log-time model of grating stability [5]. Notably, this shift cannot be related to ambient temperature fluctuations during the FBG measurements, as those were recorded at the same temperature (18 °C). Additionally, the sputter-coating process can also slightly decrease the FBG peak amplitude by ~20%. However, as shown in Figure 4a, pre-annealing the FBG at 290 °C for 3 h prior to performing the sputtering prevented this reduction in the peak amplitude. Therefore, it is highly recommended to be aware of the possible effects of the metallization process on the spectral quality of an FBG and the reflection amplitude level. For example, a severe reduction in the peak amplitude can further complicate maintaining a good signal-to-noise ratio during cryogenic sensor calibrations.

#### 3.1.2. Effect of Electrodeposition Process on FBG Spectra

**Figure 5:**
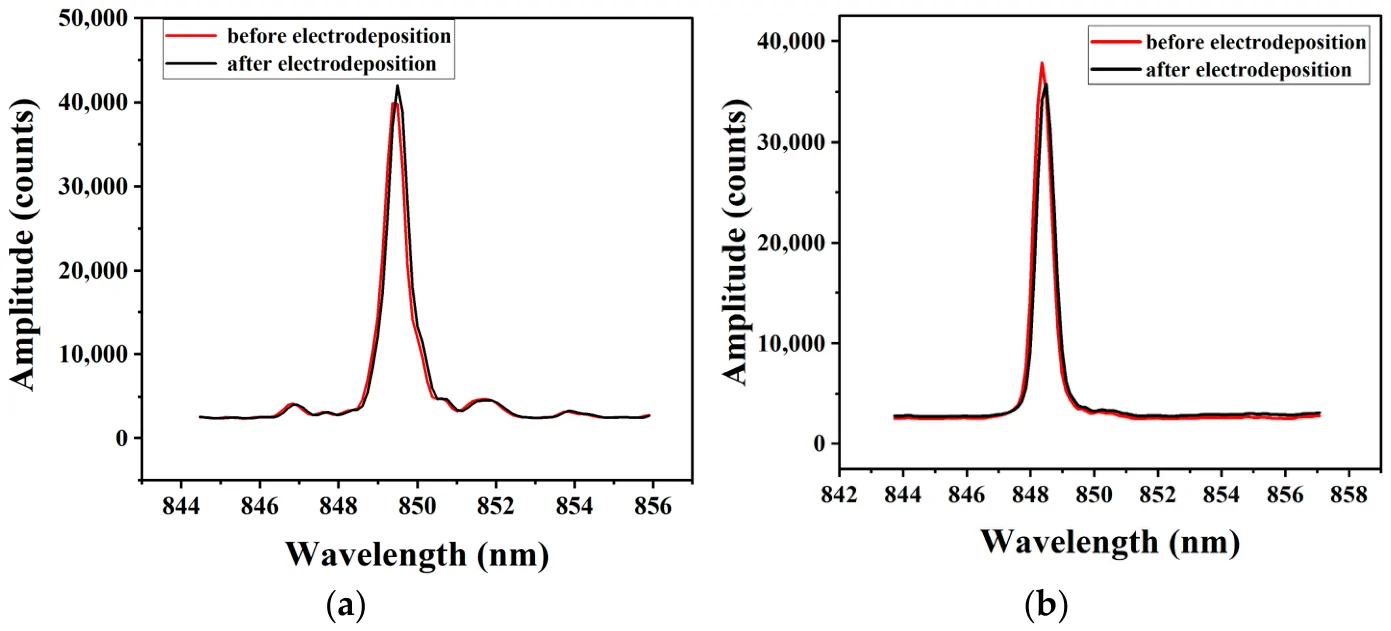
Effect of electrodeposition coating process on the FBG spectral quality for (**a**) a 585 µm thick indium coating and (**b**) a 1013 µm thick indium coating.

In contrast with sputtering, electrodeposition did not significantly affect the peak amplitude and shape of the FBGs. An average redshift of 0.14 nm in the Bragg wavelength was observed for the four investigated FBGs, with a standard deviation of 0.013 nm. Figure 5a,b illustrate the spectra of two of those FBGs with different indium coating thicknesses. Our results confirm a trend observed in the literature for zinc electrodeposition on FBGs with aluminum as a first layer [26]. This shift towards higher wavelengths is mainly due to the increase in longitudinal force exerted by the growing thickness of the secondary metal layer [26]. Furthermore, one of the most difficult challenges facing FBG metal functional coatings is related to the potential birefringence issue caused by the process, which typically manifests as spectral splitting [26]. This can complicate or even make impossible Bragg peak detection and real-time tracking. For example, in another study [27], nickel electrodeposition was reported to cause significantly larger blue shifts (~4 nm), spectral splitting, significant peak reduction, and bandwidth broadening. Here, in our work, the absence of the birefringence issue during both sputtering and electrodeposition was likely achieved thanks to good control of the high radial stress homogeneity and a sufficiently symmetrical coating thickness around the FBGs.

### 3.2. Effect of Bonding Material and Fixing Techniques on Copper–Indium Bimetallic-Coated FBG Calibration

In this section, we experimentally characterize and discuss how varying the bonding materials and fixing techniques affected the thermal stabilization and calibration accuracy of the same 585 ± 20 µm thick layer indium-coated FBG.

#### 3.2.1. Hysteresis Effect

**Figure 6:**
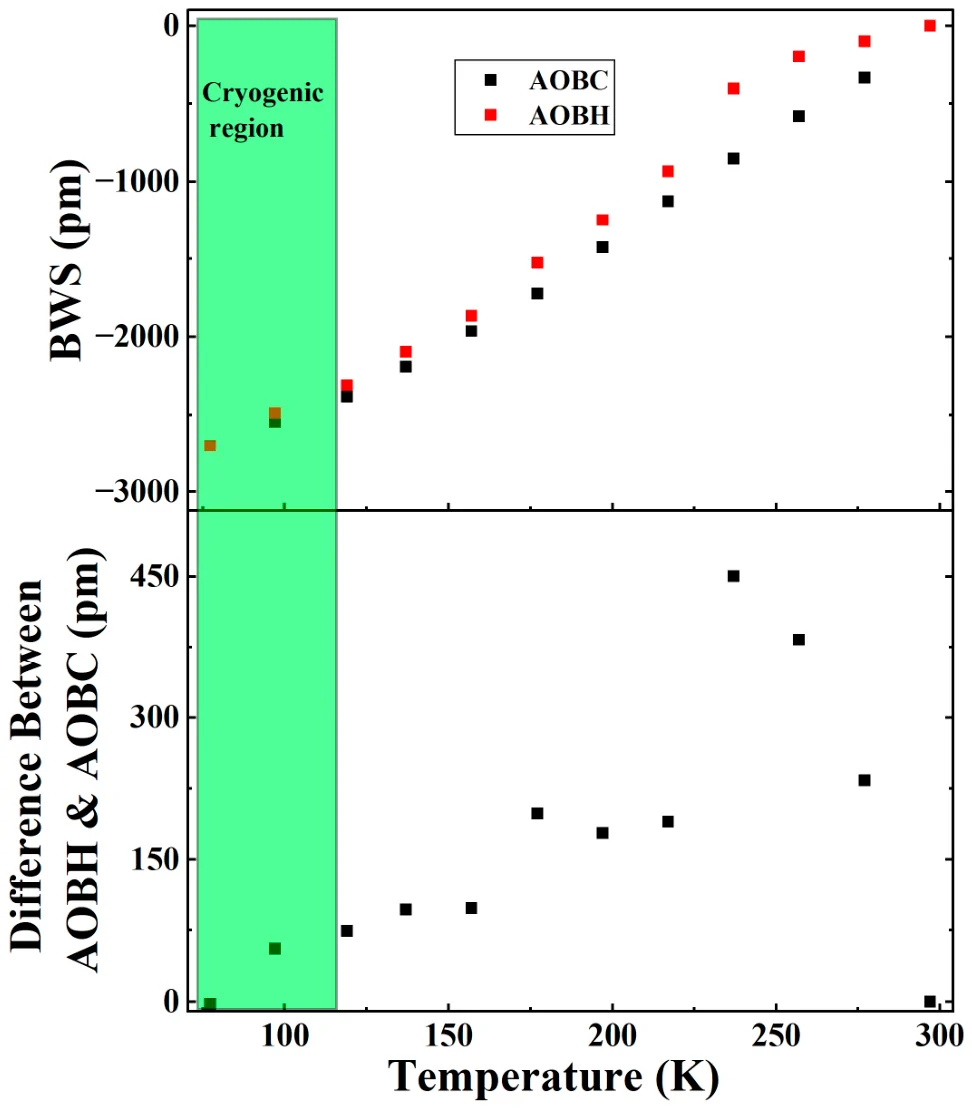
Hysteresis effects. (**Top**): AOBC (Apiezon thermal grease one-sided bonding cooling cycle); AOBH (Apiezon N thermal grease one-sided bonding heating cycle test). (**Down**): Difference in BWS between the two cycles of heating–cooling.

The properties of the Apiezon N grease strongly evolve over the temperature range from 237 K to 277 K, as reported in [14]. Additionally, as shown in Figure 2c, one-sided bonding can also be associated with a small air gap between the metal-coated FBG and the cryostat sample holder, which, of course, affects the thermal coupling efficiency and then the calibration accuracy. Therefore, in this work, hysteresis effects were only investigated for the worst-case scenario that corresponded to one-sided bonding with the Apiezon N thermal grease. As shown in Figure 6, for a thermal cycle in the order of heating to cooling, the BWS was calculated by subtracting the Bragg wavelength at RT from that measured at the other investigated temperatures. In cryogenic temperature regions (77 K to 120 K), the hysteresis effects between the cooling and heating steps showed less than a 3% difference. However, for higher temperatures, specifically at 250 K, there was a ~60% discrepancy. The reason for this increase in discrepancy is not clearly understood. However, it might be due to the Apiezon N grease properties changing from around 237 K to 277 K [14,28]. Therefore, for a systematic investigation of the effects of different bonding materials, it is essential to compare calibrations using identical heating and cooling thermal cycles. These investigations are detailed in Section 3.2.2 and Section 3.2.3. The measurement error bars are not included in Figure 6, as for the subsequent plots, they are smaller than the symbol size used (mostly less than 5 pm).

#### 3.2.2. Effect of One-Sided Kapton Tape and Apiezon Thermal Grease

**Figure 7:**
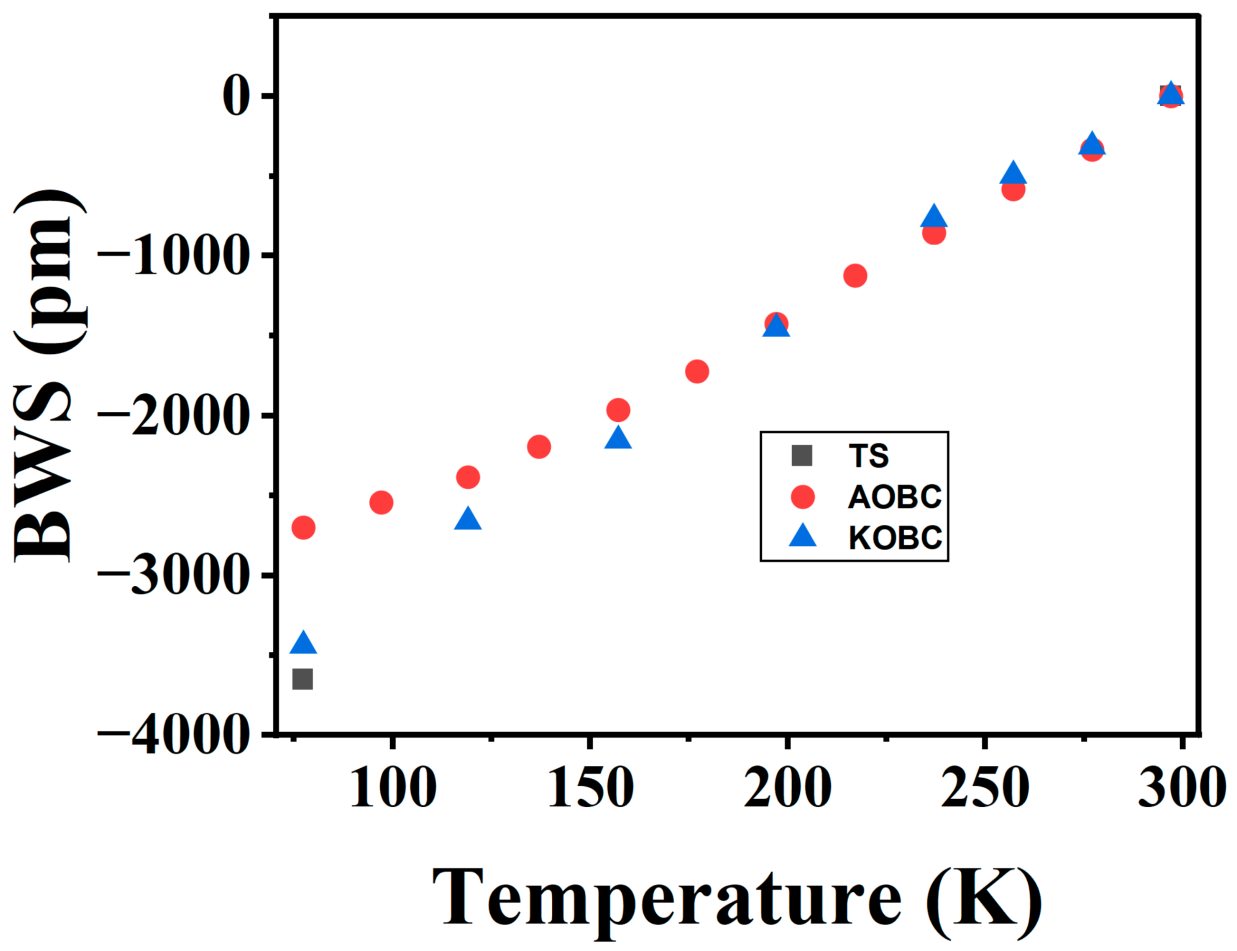
Kapton tape and Apiezon N grease one-sided bonding comparison during cooling cycle test: TS (thermal shock test), AOBC (Apiezon N thermal grease one-sided bonding cooling cycle), and KOBC (Kapton tape one-sided bonding cooling cycle test).

Figure 7 shows that for temperatures from room temperature down to 150 K, there was no significant difference in the Bragg wavelength shift between the Kapton tape and Apiezon N grease bonding materials. However, the difference in the BWS became significant at cryogenic temperatures. For example, this difference reached 33% at 77 K. This could be explained by the different thermal coupling efficiencies of these two bonding materials. Furthermore, for the Kapton tape case, the copper–indium-coated FBG was directly in contact with the sample holder, whereas for the Apiezon N grease material, the grease could act as a thermal buffer layer, preventing the direct thermal contact of the metal-coated FBG with the holder and then, of course, impacting its thermal stability. Moreover, an additional thermal shock test was carried out by plunging the coated FBG directly into a liquid-nitrogen-containing dewar to compare the associated BWS with that obtained with one-sided bonding Kapton and Apiezon N thermal grease. With this test, we could ensure that the sensor was perfectly in contact with the liquid nitrogen. At liquid-nitrogen temperature, the BWS with one-sided Apiezon N thermal grease was ~0.9 nm lower than the one obtained by the thermal shock test. In these cryogenic regions, the comparison between the different results confirmed that the Kapton tape allowed better thermal contact than the grease, as the associated BWS was closer to the one obtained through the thermal shock measurement.

Additionally, using the same surface area for the grease, a Kapton tape one-sided bonding heating cycle was attempted, and the sensor was found fully detached from the cryostat’s vertically aligned sample holder, likely due to mechanical slippage induced by the continuous depressurization of the vacuum system. In order to avoid such technical issues, a balance should be struck among the Kapton tape surface area of bonding to the metal-coated section of the fiber, thermal coupling, and mechanical integrity of such sensors on their vertical holder without introducing any external unwanted mechanical stress to the sensor during bonding processes.

#### 3.2.3. Effect of Kapton Tape and Apiezon N Thermal Grease Full Bonding

**Figure 8:**
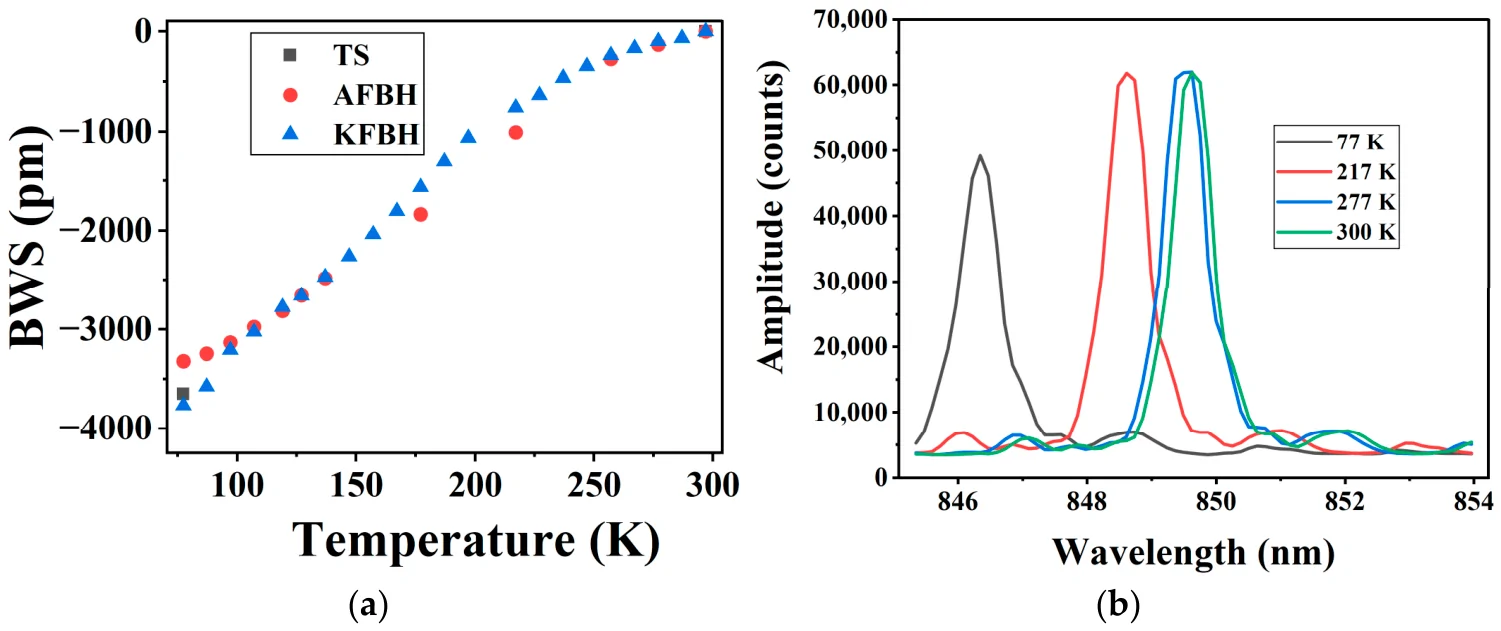
(**a**) Kapton tape and Apiezon thermal grease full-bonding comparison during heating cycle test: KFBH (Kapton tape full-bonding heating cycle test), AFBH (Apiezon N thermal grease full-bonding heating cycle), and TS (thermal shock test). (**b**) Typical spectra at different temperatures with Apiezon N thermal grease full bonding.

The magnitude of the BWS was slightly higher during full-bonding techniques thanks to better thermal contact between the metal-coated FBG and the gold-plated cryostat sample holder. For example, Figure 8a shows that at 77 K, the difference in the BWS reached 24% between the two different bonding materials. To avoid unwanted external stress to the FBGs during the bonding procedures, the results were compared with those of the thermal shock tests. This way, it was confirmed that there were no significant BWS differences at this particular temperature, ensuring the quality of the bonding. Additionally, as shown in Figure 8b, with the Apiezon N thermal grease bonding, the amplitude of the Bragg peak at 77 K was only reduced by around 19% with respect to its RT value. The origin of this reduction is not clearly understood, but it could probably be related to some bending losses within the cryostat. The impact of this effect on the quality of the measurement remains negligible as long as the reflection peak for the FBG is sufficiently high (>12 dB in our case) for the selected interrogator. The Bragg peak, as expected, showed a red shift of ~3.4 nm due to the temperature shift from liquid-nitrogen temperature to RT, which was around 0.3 nm lower than the results obtained by the thermal shock test.

## 4. Discussion

The two-step metallization processes used for our type I FBGs, based on sputtering and electroplating techniques, had no significant impact on the quality of the metallized FBGs, enabling effective real-time monitoring of the Bragg peak using commercial interrogators. However, our study confirms that the nature of the bonding materials and the chosen bonding methods can have a greater impact on the FBGs’ temperature response in cryogenic and vacuum environments. The bonding step could then be optimized to offer better thermal stability and calibration accuracy, as demonstrated in Section 3.2.2 and Section 3.2.3. As an example, our results show that the Kapton tape provided a better and more stable thermal coupling than the Apiezon N thermal grease. Similarly, in [20], this tape was also found to be a better choice that meets the same type of needs as silicone-based bonding materials.

**Figure 9:**
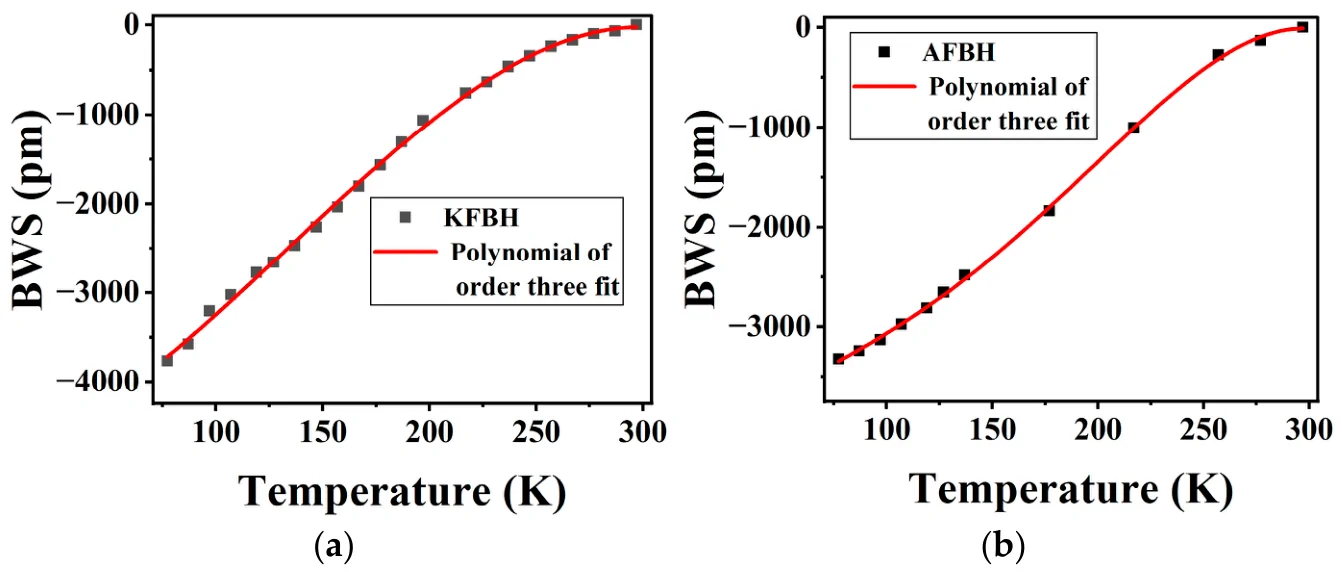
Curve fittings for BWS vs. temperature for Kapton tape and Apiezon N thermal grease bonding material: (**a**) Kapton tape full bonding; (**b**) Apiezon N thermal grease full bonding.

The thermal sensitivity of FBGs bonded with those materials is further analyzed for comparison purposes. Following Figure 8, the BWS (pm)–temperature (K) dependencies can be well-fitted by the third-order polynomials expressed in Equations (3) and (4) with R^2^ values of 0.9984 and 0.9989 for Kapton tape and Apiezon thermal grease bondings, respectively, as shown in Figure 9.

BWS (pm) = −2.7918 × 10^−4^ × T^3^ + 0.1125 × T^2^ + 7.4101 × T − 4833.3583 (3)

BWS (pm) = −3.7373 × 10^−4^ × T^3^ + 0.2026 × T^2^ − 16.8804 × T − 3047.6772 (4)

From these fitted responses, the sensitivity at a temperature (T) can be calculated by simple mathematical differentiation with respect to temperature (T).

**Figure 10:**
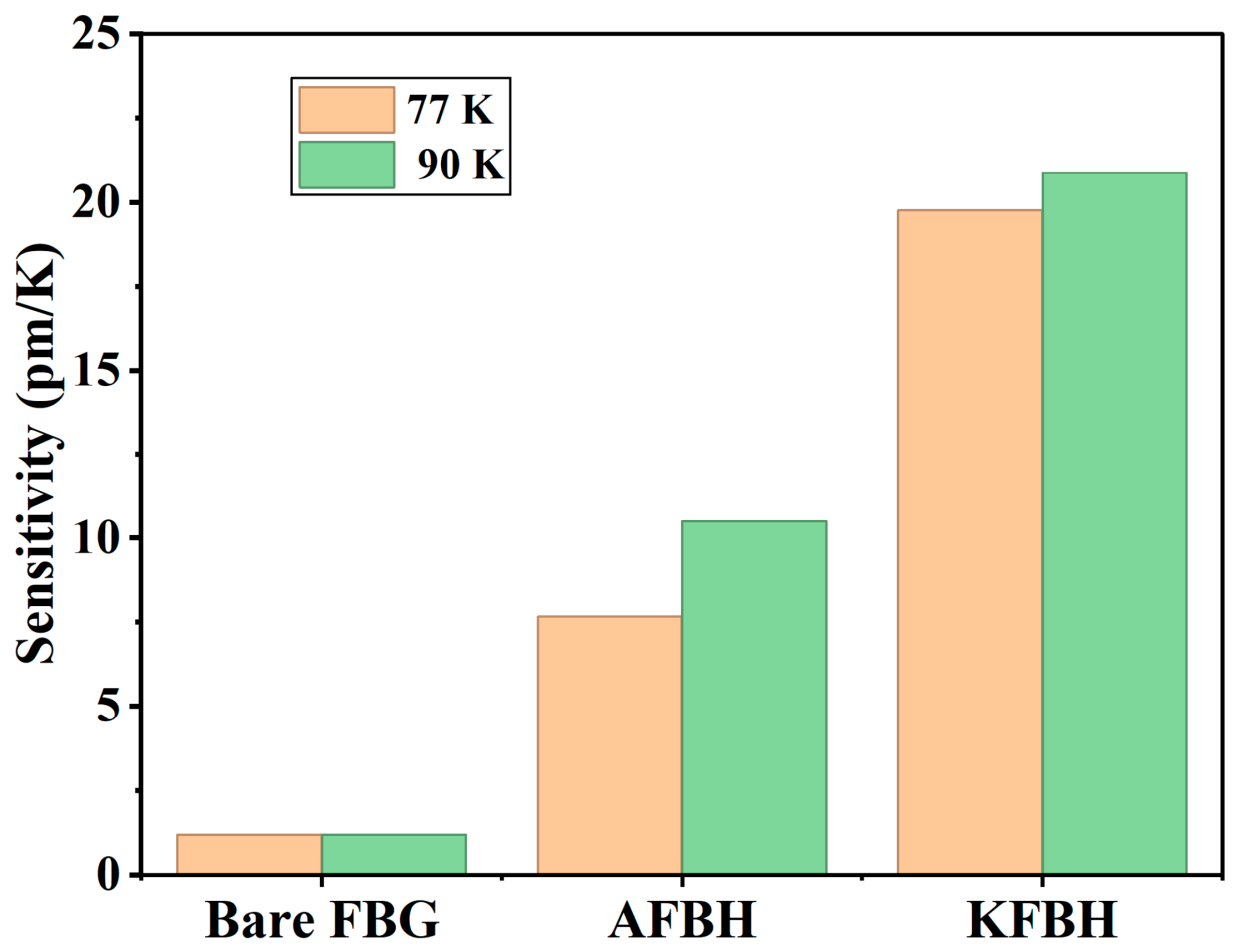
Bare and copper–indium bimetallic-coated FBG sensitivity comparison when bonded (full bonding) with Kapton tape and Apiezon N thermal grease at liquid-nitrogen (77 K) and liquid-oxygen (90 K) temperatures.

Figure 10 compares the thermal sensitivity coefficients at liquid-nitrogen (77 K) and liquid-oxygen (90 K) temperatures of bare FBGs (estimated at 1.1 ± 0.2 pm/K at 850 nm from [24]) and copper–indium bimetallic-coated FBGs with both Kapton tape and Apiezon N thermal grease full-bonding materials. The sensitivity achieved at these temperatures with Kapton tape bonding can be ~2.5 and two times higher than that achieved with Apiezon N thermal grease at 77 K and 90 K respectively. With respect to the bare FBGs, the developed bimetallic-coated FBGs with full Apiezon and Kapton tape bonding enhanced the thermal sensitivity of the bare sensors by ~8 and 17 times, respectively.

## 5. Conclusions

This work described the influence of manufacturing processes on the performance of FBGs coated with a copper–indium bimetallic layer. These FBG functional material coatings were developed using sputtering- and electrodeposition-based micromanufacturing techniques to enhance their cryogenic thermal sensitivities. The presented process allows the manufacturing of FBGs optimized for an 850 nm interrogation wavelength. The quality of the FBG spectra was not significantly affected by the addition of the two surrounding coatings. We then investigated the effect of bonding materials such as Kapton tape and Apiezon N thermal grease, varying the technique to bond the FBG to the cryostat sample holder. In the cryogenic temperature region, Kapton tape with the full-bonding fixing technique can lead to higher thermal sensitivity compared with the other three fixing techniques used in this study. For the studied sensors, the coating roughness level of the functional coating material had no significant impact on the sensor performance. However, the influence of severe roughness levels in thicker coatings (higher than that of a 700 µm thick indium coating) will be the topic of a future study to see if, for such sensors, Apiezon thermal grease could be a better choice. An important result is that, with respect to bare FBGs, the developed bimetallic-coated FBGs with full Apiezon N grease and Kapton tape bondings exhibited increased thermal sensitivities by ~8 and 17 times, respectively. Finally, our study demonstrates that, both during calibration and field deployment, the fixing methods of the metal-coated FBGs must be carefully chosen to ensure optimal thermal coupling between the sensor and the target environment or equipment. The developed sensor performance must be investigated down to liquid-helium temperature to cover the full range of cryogenic applications. Additionally, the introduction of another metal seed layer might have an impact on the sensor performance, as reported in [29]. Of course, this depends on the type of metal and its compatibility, so this will be addressed in our future study.

## Data Availability

The original contributions presented in this study are included in the article. Further inquiries can be directed to the corresponding authors.

## Abbreviations

The following abbreviations are used in this manuscript:

AFBH	Apiezon thermal grease full-bonding heating cycle
AOBC	Apiezon thermal grease one-sided bonding cooling cycle
AOBH	Apiezon thermal grease one-sided bonding heating cycle test
BWS	Bragg wavelength shift
Cu	Copper
FBG	Fiber Bragg grating
KOBC	Kapton tape one-sided bonding cooling cycle test
KFBH	Kapton tape full-bonding heating cycle test
RT	Room temperature
TS	Thermal shock test
UV cw	Ultraviolet continuous wave
